# First EST-SSRs of *Helichrysum italicum* (Roth) G. Don (Asteraceae) Revealed Insights into the Genetic Diversity and Population Structure in Corsica

**DOI:** 10.3390/plants14243794

**Published:** 2025-12-12

**Authors:** Petra Gabrovšek, Matjaž Hladnik, Dunja Bandelj, Zala Jenko Pražnikar, Saša Kenig, Félix Tomi, Marc Gibernau, Slavko Brana, Alenka Baruca Arbeiter

**Affiliations:** 1 Faculty of Mathematics, Natural Sciences and Information Technologies, University of Primorska, Glagoljaška 8, 6000 Koper, Slovenia; petra.gabrovsek@upr.si (P.G.); matjaz.hladnik@upr.si (M.H.); dunja.bandelj@upr.si (D.B.); 2 Faculty of Health Sciences, University of Primorska, Polje 42, 6310 Izola, Slovenia; zala.praznikar@fvz.upr.si (Z.J.P.); sasa.kenig@fvz.upr.si (S.K.); 3 Laboratoire Sciences Pour L’Environnement, Université de Corse-CNRS, UMR 6134 SPE, Route des Sanguinaires, 20000 Ajaccio, France; tomi_f@univ-corse.fr (F.T.); gibernau_m@univ-corse.fr (M.G.); 4 Istrian Botanical Society, Trgovačka 45, 52215 Vodnjan, Croatia; istra.botanica@gmail.com

**Keywords:** *Helichrysum italicum*, immortelle, de novo transcriptome, genic microsatellite markers, EST-SSRs, population study, Corsica, Mediterranean Basin

## Abstract

*Helichrysum italicum* (Roth) G. Don (Asteraceae) is a valuable medicinal and aromatic plant native to a variety of habitats across the Mediterranean region. However, genetic studies of this morphologically diverse species have been limited by the scarcity of species-specific DNA markers. To address this limitation, we generated the first de novo transcriptome assembly comprising 24,806 transcripts from young shoots containing leaves and flowers, developed EST-SSR markers, and evaluated their utility in population genetic analysis. Seventy-eight primer pairs were designed, of which 23 showed successful amplification, polymorphism, and transferability to *Helichrysum litoreum* Guss. and *Helichrysum arenarium* (L.) Moench. A subset of 12 EST-SSRs was used to genotype 270 individuals from 12 natural populations of *H. italicum* in Corsica (France), along with one outgroup population from Croatia. The polymorphic information content ranged from 0.250 to 0.796, and Shannon’s information index ranged from 0.588 to 1.843, indicating the markers’ suitability for population genetic studies. Analysis of molecular variance revealed that 15% of the total genetic variation was attributable to differences among populations. Discriminant analysis of principal components and Bayesian clustering in STRUCTURE identified distinct population clusters corresponding to geographic locations. Notably, the southernmost coastal populations were clearly differentiated from the others.

## 1. Introduction

*Helichrysum italicum* (Roth) G. Don (Asteraceae), commonly known as “immortelle” or “everlasting flower” [1,2], is a characteristic plant of the Mediterranean xerophilous shrub vegetation. It is very adaptable from a phytosociological perspective and forms plant communities associated with different habitat types—from coastal sand dunes and rocky cliffs to dry karst meadows and sunny slopes with maquis’ vegetation. In many of these plant communities, it occurs as a dominant or characteristic species [3,4,5,6,7], which emphasizes its ecological importance and the potential vulnerability of plant communities in the Mediterranean islands due to environmental conditions [4,8].

*H. italicum* is a valued medicinal and aromatic plant whose aerial parts have been traditionally used for centuries in folk medicine to treat digestive disorders, skin inflammations, and respiratory diseases [1]. The essential oil of *H. italicum*, synthesized in specialized epidermal glandular trichomes of the aerial parts of the plant [9], has a very complex chemical composition dominated by monoterpenes (α-pinene, limonene, nerol, neryl acetate) and sesquiterpenes (α-selinene, β-selinene, γ-curcumene, trans-β-caryophyllene) [10], as well as β-diketones, most notably italidiones [11]. Furthermore, the aqueous and alcoholic extracts of *H. italicum* contain a wide range of phenolic compounds, including flavonoids, phenolic acids and acetophenones [2,10].

Nowadays, the essential oil of *H. italicum* is highly sought after in the cosmetics industry for its anti-aging properties, including stimulation of skin cell regeneration, improved skin texture, and reduction of fine lines and wrinkles [12]. Among its bioactive compounds, neryl acetate plays a key role by exerting a significant effect on the keratinocyte differentiation process and promotion of skin barrier formation [13], which helps prevent excessive water loss and protects the body against mechanical, microbial, and oxidative insults [14]. Compounds such as α-pinene and limonene exhibit inhibitory activity against collagenase and elastase, enzymes associated with the skin-aging process [15], while italidiones act as anti-inflammatory agents and protect the skin against UV radiation [16]. *H. italicum* has attracted growing interest also from the pharmaceutical and food industries due to its scientifically confirmed therapeutic properties, including antimicrobial [17,18,19,20,21,22,23,24], antioxidant [25,26], anti-inflammatory [27,28], insecticidal [29,30,31,32], and antiviral [25,33,34] effects and cytotoxic activities against some human cancer cell lines [35,36].

To meet industry and market demands, *H. italicum* plantations have been established across many Mediterranean countries [11,37]. The cultivation of medicinal and aromatic plants not only addresses current and future demands for large-scale production of plant-based drugs and herbal remedies but also helps reduce excessive harvesting from natural populations for commercial purposes and contributes to the preservation of natural habitats [38]. Overexploitation of wild plants in their natural sites has been noted especially along the eastern Adriatic coast, and its impact on overall genetic diversity is expected to become apparent in the future [39]. The main cultivation areas of *H. italicum* are located in the northwestern Mediterranean, especially in southern France, including Corsica, and in Italy, where the plant has a long-standing tradition, is deeply rooted in the landscape and the cultural heritage, and is also used in various cosmetics [40,41]. In Corsica, its cultivation increased markedly during the decade between 2010 and 2020, with both a change in the number of farmers (from 50 to 125) and the expansion of planted surfaces (from 214 to 618 ha) [42]. The Corsican essential oil of *H. italicum* is highly valued and considered a premium product on the global market due to its particular property of high neryl acetate and italidiones content [13,43,44], which remains the benchmark of quality in the cosmetics industry [41]. There are also two recognized subspecies of *H. italicum* present in Corsica: the widespread subsp. *italicum* and the subsp. *tyrrhenicum*, which is restricted to the southern part of the island [45]. The distribution of these subspecies often overlaps, and consequently, many individuals with intermediate morphological traits [45,46] and chemical profiles can be observed, potentially influencing the biological activities of plant extracts [24].

One of the major challenges in *H. italicum* cultivation is the selection of appropriate plant material for large-scale production in order to obtain commercially valuable plants for industrial use. Growers usually obtain the seedlings directly from wild populations, which results in highly diverse and inconsistent plant material. The species is characterized by a pronounced morphological and chemical plasticity, observed not only between distant populations but also within populations from the same geographical region [17,47]. The combined morphological variability and chemical heterogeneity poses a challenge for the reliable identification of species/subspecies. The absence of adequate control and certification systems has, in some cases, led to economic losses—primarily due to the production of essential oils with unsuitable chemical profiles or insufficient levels of biologically important compounds demanded by the industry [48,49]. Furthermore, superior, completely characterized commercial cultivars of *H. italicum* have not yet been registered, indicating concerns about the quality of raw plant material for industrial applications [39]. The domestication and systematic cultivation of *H. italicum* therefore require clear identification of the genetic material to reduce the possibility of plant misidentification and adulteration and to ensure consistent quality and stable yields of valuable secondary metabolites in essential oils and other extracts for industrial use [48].

Most published studies on *H. italicum* have addressed the phytochemical composition of plant extracts and their medicinal properties. Genomic studies somewhat lag behind—most likely due to the limited number of species-specific molecular markers. Recently, the whole genome sequencing (WGS) approach was used to generate genomic sequences of *H. italicum* subsp. *italicum*, which were subsequently employed for chloroplast genome assembly [50]. The assembled nuclear DNA contigs and chloroplast genome served as sources for the development of the first *H. italicum* specific microsatellite markers (SSRs), including 24 genomic SSRs [48] and 16 chloroplast SSRs [49]. The transferability of both marker sets was also confirmed for the closely related species *H. litoreum* Guss. and *H. arenarium* (L). Moench [48,49]. Furthermore, the genomic SSR markers were successfully applied in a conservation biology approach to develop a strategy for the genetic restoration of *H. arenarium*, a threatened species in Europe with only a few remaining natural populations [51].

The plant genotype, together with the environmental factors, plays a key role in the synthesis of biologically active compounds [52]. Therefore, the development and comprehensive analysis of transcriptomes of medicinal and aromatic plants (MAPs) is of particular importance. Large-scale RNA sequencing (RNA-seq) enables functional gene mining and the identification of biosynthetic pathways for secondary metabolite while providing a great opportunity for the development of genic SSR markers (EST-SSRs) [53,54,55]. Deriving from expressed genes, EST-SSRs are generally less variable than are genomic SSR markers, a characteristic that may increase their utility in distinguishing species and subspecies [56]. Several studies have highlighted the advantages of EST-SSR markers in MAPs, which have been successfully used in related species within the genus *Salvia* [57], in the assessment of genetic diversity across multiple ecotypes and cultivars in the genus *Ocimum* [58], and in conservation studies and chemotypes discrimination in *Mentha piperita* [59]. The use of EST-SSRs enables plant breeders to screen large plant populations [55] and represents a prerequisite for targeted breeding programs and the development of commercial cultivars of *H. italicum* [39].

To address the challenges outlined above, it is essential to understand the genetic structure and diversity of *H. italicum* across agro- and natural ecosystems. Such knowledge would support the selection of genotypes to ensure consistent quality and stable yields of bioactive metabolites, strengthen traceability systems to prevent adulteration, aid in the conservation of natural populations, and clarify relationships within and between subspecies. Identifying distinct genetic groups can also reduce the risk of misidentification and guide the selection of appropriate germplasm for cultivation.

Therefore, the objectives of this study were to (a) generate the first transcriptome assembly of *H. italicum* subsp. *italicum*, (b) perform functional annotation, (c) develop the first set of EST-SSR markers for *H. italicum* subsp. *italicum*, (d) evaluate the cross-transferability of the developed EST-SSRs to closely related species *Helichrysum litoreum* Guss. and *Helichrysum arenarium* (L.) Moench, and (e) apply the newly developed markers to assess the genetic structure of natural populations of *H. italicum* in Corsica (France).

## 2. Results

### 2.1. Transcriptome Sequencing and De Novo Assembly

The de novo transcriptome assembly was constructed by extracting mRNA from young shoots with leaves and flower buds, sampled over six phenological stages and pooled prior to sequencing. In total, 21,568,881 raw reads were obtained, with an average length of 175 bp, corresponding to 3.78 Gb, and a GC content of 42%. The number of assembled transcripts and their length varied among the assemblies (Table 1). The results showed that the rnaSPAdes generated longer transcripts compared to MIRA, with an average length of 1498 and 1832, using shorter and longer k-mers, respectively. Additionally, *H. italicum* transcriptome assemblies were assessed based on their orthologue’s completeness by the BUSCO approach, and quality evaluation of completeness was ranked according to the following order: rnaSPAdes (77), 49.2%; rnaSPAdes, (127) 48.6%; and MIRA, 43.6%. Both rnaSPAdes assemblies were similar based on evaluated BUSCO completeness and outperformed the MIRA assembly. The rnaSPAdes (127) assembly was selected as the final assembly because it contained fewer duplicated BUSCOs than did the rnaSPAdes (77) assembly. It comprised 24,806 contigs, with a N50 of 2030 bp and a total length of 45,445,976 nt.

To assess the selected assembly, TMAP software was used to map reads to the transcripts, and 93.82% of the reads mapped to the assembly. TPM (transcripts per million reads) was determined for each transcript using SALMON. As all transcripts had a TPM value above 0.5, and all were included in the functional annotation and biological classification.

### 2.2. Functional Annotation and Biological Classification

The assembled transcripts were utilized for the functional annotation analysis. Firstly, taxonomic classification was conducted primarily as contamination control. Based on the classification obtained by the MMSeqs2 taxonomy method against NCBI nr database, most reads were assigned to the family Asteraceae (84.8%). Within Asteraceae the most frequent species-level assignments were *Erigeron canadensis*, *Cynara cardunculus*, *Helianthus annuus*, and *Artemisia annua* (Table 2). Only 68 transcripts were assigned to other taxonomic kingdoms and were therefore removed.

Annotation against the NCBI nr, RefSeq protein, and UniProtKB/Swiss-Prot databases returned significant hits for 99.5%, 99.1%, and 89.0% of the transcripts, respectively (Table 3). GO, COG, KOG, and EggNOG terms were further assigned from the EggNOG database to 62.5%, 50.0%, 68.3%, and 98.6% of the transcripts, respectively. Finally, 55.4% of the transcripts were annotated with KEGG Orthology (KO) identifiers.

**Figure 1:**
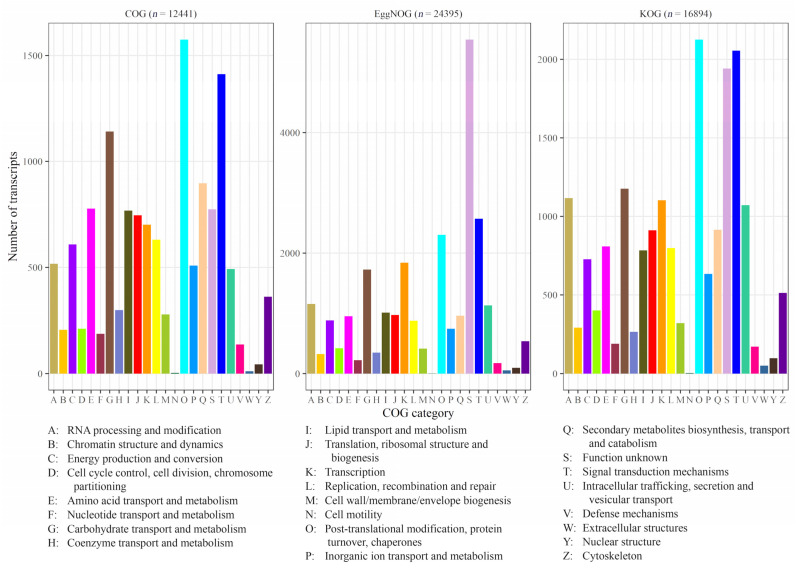
EggNOG with COG and KOG classifications of *H. italicum* transcripts.

Annotations from COG, EggNOG, and KOG were each classified into 24 functional categories (Figure 1). Across all three databases, the dominant categories, excluding category S (‘function unknown’), were ‘post-translational modification, protein turnover, chaperones’ and ‘signal transduction mechanisms’, each accounting for 9% to 11% of the annotated transcripts. In the COG classification, these categories were followed in frequency by ‘carbohydrate transport and metabolism’ (1141; 8.6%) and ‘secondary metabolites biosynthesis’ (897; 6.7%), whereas in the KOG classification, they were followed in frequency by ‘carbohydrate transport and metabolism’ (1177; 6.4%), ‘RNA processing and modification’ (1117; 6.0%), and ‘transcription’ (1103; 6.0%). The distribution of the most represented functional categories in EggNOG was similar to that observed in KOG. The less represented functional groups but those important for eukaryotes were ‘defense mechanisms’, ‘nucleotide transport and metabolism’, ‘chromatin structure and dynamics’, and ‘coenzyme transport and metabolism’ (representing from 0.7% to 1.4% and 0.9% to 1.6% according to the EggNOG and KOG classifications, respectively).

**Figure 2:**
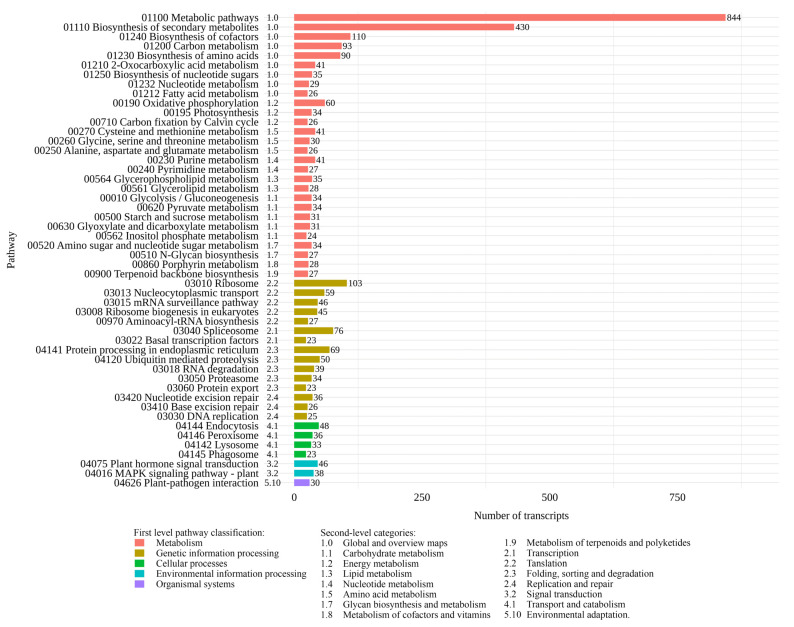
KEGG classification of *H. italicum* transcripts. The top 50 plant-related pathways are shown, sorted according to the KEGG first- and second-level pathway organization.

To identify active biological pathways in young shoots with leaves and flower buds of *H. italicum*, 13,711 transcripts with orthologs in the KEGG orthology database were classified into five major pathway categories. The largest proportion of transcripts was assigned to metabolism, followed by genetic information processing, cellular processes, environmental information processing, and organismal systems (Figure 2). Within the second-level category, ‘1.0 Global and overview maps’, most transcripts were associated with metabolic pathways (01100) and biosynthesis of secondary metabolites (011100). Among other second-level categories, oxidative phosphorylation (00190) from the ‘1.2 Energy metabolism’ was most highly represented, followed by photosynthesis (00195). Pathways from the group ‘1.10 Biosynthesis of other secondary metabolites was also observed in the annotation results, albeit with a smaller number of transcripts (e.g., phenylpropanoid biosynthesis (00940), biosynthesis of various plant secondary metabolites (00999) and flavonoid biosynthesis (00941), which were linked to 15, 13, and 9 transcripts, respectively). The genetic information processing category was predominantly represented by pathways such as ribosome (03010) (‘2.2 Translation’), spliceosome (03040) (‘2.1 Transcription’), and protein processing in endoplasmic reticulum (04141) (‘2.3 Folding, sorting, and degradation’). In the second-level KEGG categories, ‘4.1 Transport and catabolism’, ‘3.2 Signal transduction’, and ‘5.10 Environmental adaptation’, the majority of transcripts were mapped to the pathways of endocytosis (04144), plant hormone signal transduction (04075), and plant–pathogen interaction (04626), respectively.

**Figure 3:**
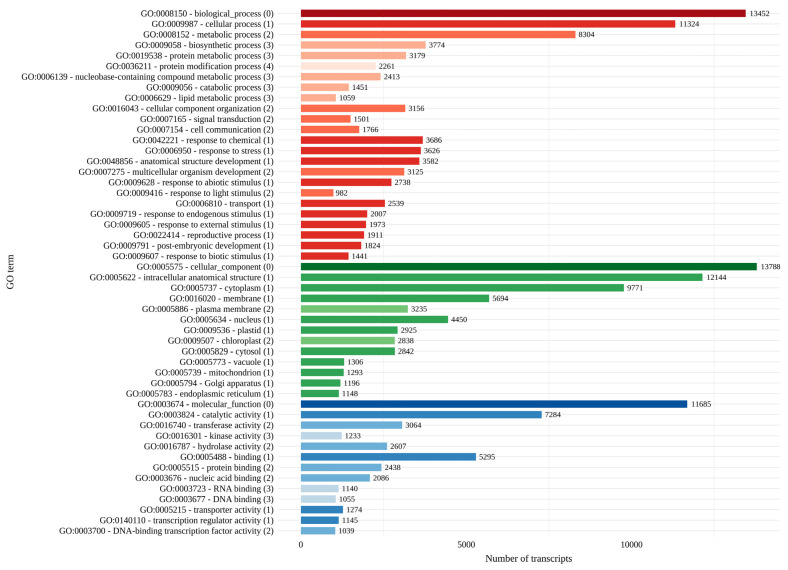
The 50 most represented plant GO slims based on the classification of *H. italicum* transcripts. Numbers in parentheses and color shades indicate the GO level, whereas colors indicate GO aspects as follow: biological process = red, cellular component = green, and molecular function = blue.

Mapping of GO terms to plant GO slims resulted in the assignment of 15,453 transcripts (62.5%) to the following Gene Ontology categories: biological process (13,452), molecular function (11,685), and cellular component (13,788). Within biological processes, the most represented level-1 terms were cellular process (GO:0009987) with several biological process on the second, third, and fourth level, followed by response to chemical (GO:0042221), response to stress (GO:0006950), and anatomical structure development (GO:0048856). GO terms related to interactions of plants to environmental stimuli (such as response to abiotic stimulus (GO:0009628) with the nested term being response to light stimulus (GO:0009416) or response to biotic stimulus (GO:0009607)) and reproduction (e.g., reproductive processes (GO:0022414)) were also among the 50 most represented processes. For molecular functions, the predominant level-1 categories included catalytic activity (GO:0003824), binding (GO:0005488), transporter activity (GO:0005215), and transcription regulator activity (GO:0140110). Among the cellular component subcategories, the most abundant terms were intracellular anatomical structure (GO:0005622), cytoplasm (GO:0005737), membrane (GO:0016020), nucleus (GO:0005634), and plastid (GO:0009536), including the nested term chloroplast (GO:0009507) (Figure 3).

### 2.3. Frequency, Characterization, and Distribution of Microsatellite Repeats in H. italicum Transcriptome

**Figure 4:**
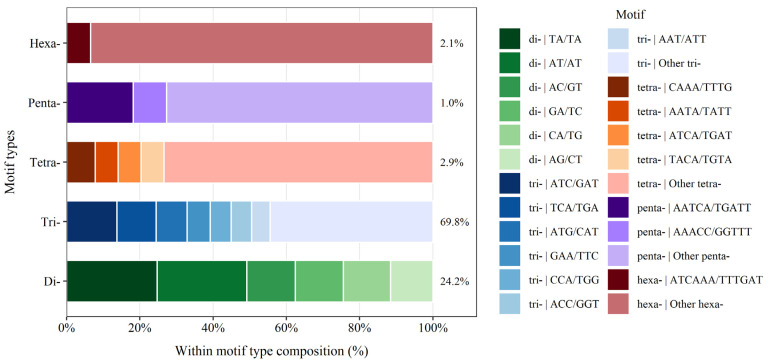
Frequency of identified microsatellite motif classes in the *H. italicum* unigenes showing within motif type composition and total abundance of each motif type (%).

The obtained transcripts were clustered into 19,921 unigenes, which were further searched for the presence of microsatellite repeats (SSRs). The analysis revealed a total number of 2107 microsatellite loci (excluding mononucleotide and compound microsatellites) (Table 4). The most abundant microsatellite motifs within *H. italicum* were trinucleotides with a frequency of 69.8% (1544), followed by dinucleotides (24.2%; 535), while tetranucleotides (2.9%; 64), hexanucleotides (2.1%; 46), and pentanucleotides (1.0%; 22) were rare. The most frequent dinucleotide repeat motif was TA/TA (24.7%), followed by AT/AT (24.5%) and AC/GT (13.3%). Among the trinucleotide repeats units, ATC/GAT, TGA/TCA, and ATG/CAT were the most common with frequencies of 13.7%, 10.7%, and 8.5%, respectively (Figure 4).

### 2.4. EST-SSR Marker Development and Cross-Amplification in Helichrysum Species

From 19,921 identified unigenes containing 2107 perfect microsatellites, primer pairs were successfully designed for 1661 SSR-containing unigenes. Further multiple sequence alignment (MSA) of unigenes with genomic DNA and the corresponding mRNA of the most closely related species confirmed that 78 loci had both primer sites located within the same exon, thereby increasing the probability of successful amplification. All of these loci were assigned putative functions based on protein and KEGG annotations.

PCR evaluation of these 78 EST-SSR loci revealed that 38.5% (30 EST-SSRs) produced clear and scorable fragments, including seven monomorphic loci, while 53.8% (42 EST-SSRs) generated ambiguous fragments, and 7.7% (6 EST-SSRs) failed to amplify. Among the 23 newly developed polymorphic EST-SSRs, most had trinucleotide motifs (87%), with two dinucleotide and one hexanucleotide motif. According to RefSeq annotations, 21 loci were located in coding sequences (CDS) and two in untranslated regions (UTRs) (Table 5).

Functional annotation of the SSR-associated transcripts based on literature review and UniProtKB/Swiss-Prot annotations of orthologous proteins revealed putative functions (Table 5). The identified proteins included transcription factors, enzymes, transporter proteins, and signaling components, many of which are directly or indirectly linked to metabolism and biosynthesis (e.g., serine acetyltransferase 2, GDSL esterase/lipase (involved in various processes, including stress response [60]), MYB46), homeostasis (metal tolerance protein 1), stress response/development (ABC transporter, WRKY6 [61], transcription elongation factor SPT6-like [62], mediator of RNA polymerase II transcription subunit 14 (MED14) [63], heterogeneous nuclear ribonucleoprotein [64], BIG GRAIN 1-like B [65], glucosidase II β-subunit [66], CEPR2, F-box/LRR-repeat MAX2 [67]), and reproduction (3-deoxy-manno-oculosonate cytidylyltransferase (KDSB), protein EARLY FLOWERING 5 [68], AAA-ATPase ASD, pollen receptor-like kinase 3 (PRK3) [69,70], glycerol-3-phosphate 2-O-acyltransferase 6 (GPAT6) [71], mechanosensitive ion channel protein 8 [72], and transcription factor TCP4–like [73,74]).

Cross-amplifications of the 23 polymorphic EST-SSRs produced alleles within the expected size range across all species. All markers amplified successfully in *H. arenarium* and *H. litoreum*, indicating conserved primer-annealing sites in the homologous regions and supporting their applicability in genetic studies across the *Helichrysum* genus. Following cross-amplification, eleven loci (EST-HiUP-04, EST-HiUP-05, EST-HiUP-08, EST-HiUP-09, EST-HiUP-11, EST-HiUP-15, EST-HiUP-16, EST-HiUP-17, EST-HiUP-20, EST-HiUP-22, EST-HiUP-23) exhibited low polymorphism; therefore, only loci with at least three alleles were retained for further characterization and population genetic diversity analysis in *H. italicum* (Table 6). At the end, the same set of 12 polymorphic EST-SSRs was used for comparison of allele size variation among *H. italicum*, *H. litoreum*, and *H. arenarium* (Table S1).

### 2.5. EST-SSR Marker Characterization and Genetic Diversity Study of H. italicum Populations

Basic genetic parameters for the final subset of 12 EST-SSR markers were calculated based on 270 samples of *H. italicum* from 13 natural populations (Table 6). Altogether 83 different alleles were amplified, and the number of amplified alleles per locus (N_a_) varied from five (EST-HiUP-02, EST-HiUP-10, and EST-HiUP-13) to ten (EST-HiUP-03 and EST-HiUP-12), with an average of 6.92 alleles per locus. The observed heterozygosity (H_o_) ranged between 0.256 and 1.0, with an average of 0.655, whereas the expected heterozygosity (H_e_) ranged between 0.261 and 0.820, with an average of 0.571. Polymorphic information content (PIC) varied between 0.250 and 0.796, with an average value of 0.530, and Shannon’s information index for these markers was between 0.588 and 1.843, with an average of 1.176. The loci EST-HiUP-01, EST-HiUP-02, EST-HiUP-13, EST-HiUP-14, and EST-HiUP-21 showed a statistically significant deviation from the Hardy–Weinberg equilibrium, whereas the estimated frequency of null alleles was not significant in any of the 12 EST-SSRs.

This set of developed EST-SSR markers was applied to evaluate the genetic diversity within natural populations of *H. italicum* from Corsica (France) and the Istrian peninsula (Croatia), based on several genetic diversity parameters (Table 7). Differences in the population genetic structure were highlighted. For example, the Col de Saint-Eustache population exhibited the highest mean number of alleles per locus (4.67), whereas the Pianottoli population showed the lowest (3.67). Across all populations, the observed heterozygosity (ranging from 0.567 in Tonnara to 0.742 in Col de Saint-Eustache) was higher than the expected heterozygosity (ranging from 0.477 in Corte to 0.598 in Col de Saint-Eustache). Shannon’s information index was also highest in Col de Saint-Eustache (1.129) and lowest in Pianottoli (0.878). Interestingly, private alleles were detected in about half (6 out of 13) of the studied populations of *H. italicum*. The highest number of private alleles (three) was detected in the population from Plage de l’Ovu Santu, whereas the Piana and Tonnara populations presented two private alleles each. A single private allele was observed in the populations of Conca, Col de Saint-Eustache, and Cape Kamenjak (Table 7 and Table S2).

Evidence of a recent bottleneck based on 12 EST-SSRs was tested for all studied *H. italicum* populations. Based on the Wilcoxon test, which returned *p*-values above 0.05 for all populations, there was no indication of a recent bottleneck event that would have disrupted mutation–drift equilibrium or affected their genetic diversity.

To evaluate the genetic diversity between natural populations of *H. italicum* from Corsica (France) and the Istrian peninsula (Croatia), pairwise F_st_ values (proportion of total genetic differentiation) were calculated (Table S3). The highest differentiation was observed between the populations of Corte and Pianottoli (F_st_ = 0.067), whereas the lowest differentiation was found between Conca and Col de Saint-Eustache (F_st_ = 0.013). As expected, the AMOVA revealed that only 15% of the total variance was attributable to differences among populations, while 85% was explained by variation within populations (Table 8).

**Figure 5:**
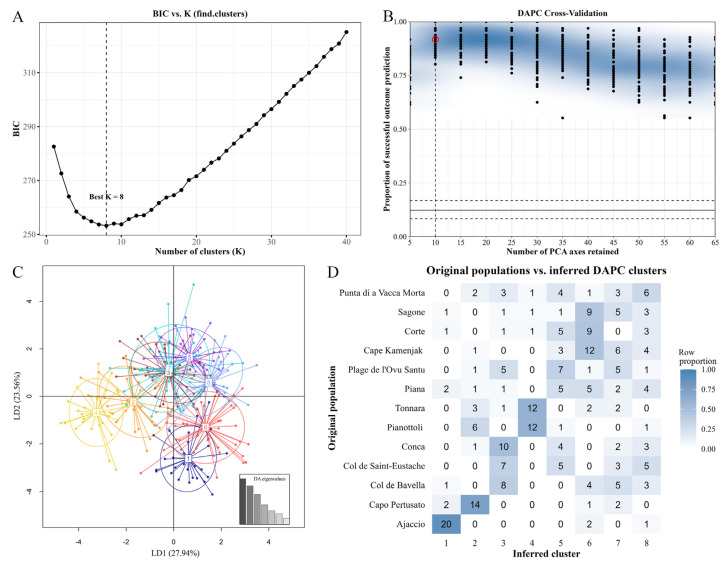
Clustering, cross-validation, and visualization of DAPC. (**A**) Bayesian inference criterion (BIC) values versus the number of clusters (K) for 270 individuals from 13 *H. italicum* populations. (**B**) DAPC cross-validation density plot for the optimal number of principal components (PCs) retained in the analysis of eight predefined groups. The red circle indicates the optimal number of PCs. (**C**) Scatterplot of DAPC of population structure, using twelve EST-SSR markers. According to the discriminant analysis function (DA) eigenvalues, the axes represent the first two linear discriminants (LD1, LD2). Each dot represents an individual, each circle represents a cluster, and clusters are numbered on the scatterplot. (**D**) Heatmap of inferred DAPC clusters versus the original populations, where the intensity of white-to-blue-colored squares is directly proportional to the number of individuals allocated to clusters.

The genetic differentiation among populations was further studied with a DAPC analysis based on the 270 individuals’ genotypes originating from the 13 natural populations of *H. italicum*. First, eight clusters (K = 8) were determined as the optimal grouping, corresponding to the lowest Bayesian information criterion (BIC = 252.7524; Figure 5A). Second, cross-validation of the number of PCA axes retained versus the proportion of successful outcome predictions indicated that 10 PCAs should be retained for subsequent analysis (Figure 5B). Hence, individuals were assigned to eight clusters and visualized using the first two linear discriminants (LD1 and LD2: 51.5% of explained variance) on a scatterplot (Figure 5C). To interpret the distribution of individuals from each population across the eight clusters, a heatmap of inferred DAPC clusters versus original populations was also examined (Figure 5D). Combined interpretation of the scatterplot and the heatmap suggested that clusters 1, 2, 4, and 6 represent distinct genetic groups. Cluster 1 consisted primarily of individuals from the Ajaccio population; in cluster 2, samples from Capo Pertusato dominated; samples from Col de Bavella, Col de Saint-Eustache, and Conca dominated in cluster 3; and individuals from Pianottoli and Tonnara were most abundant in cluster 4. Most individuals in cluster 6 were from Cape Kamenjak, followed by Sagone and Corte. In contrast, clusters 5, 7, and 8 contained mixed individuals, predominantly from Punta di a Vacca Morta, Plage de l’Ovu Santu, Col de Saint-Eustache, Conca, and Col de Bavella.

**Figure 6:**
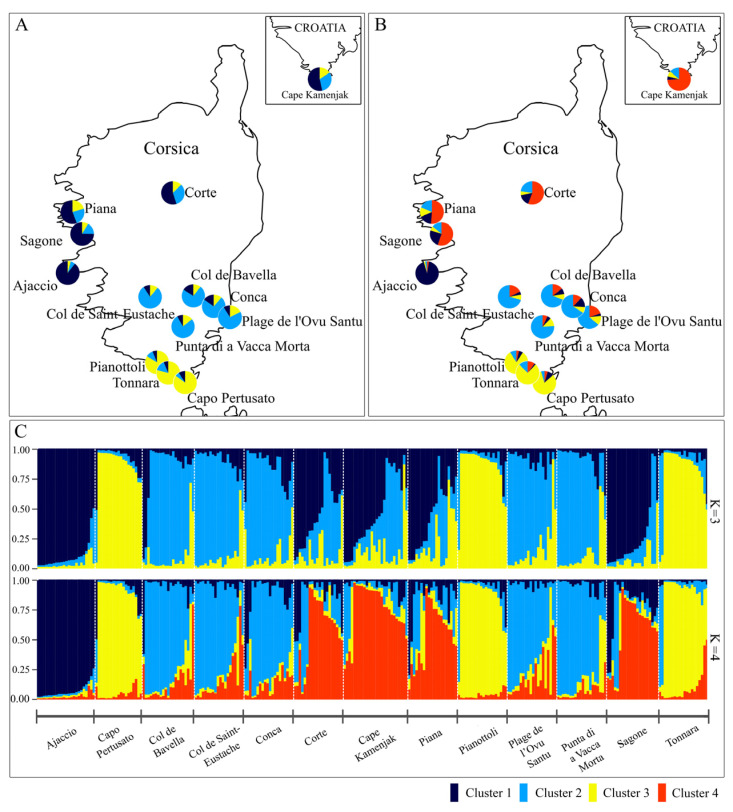
Results of the Bayesian-model-based clustering STRUCTURE analysis for 13 *H. italicum* populations (270 samples) evaluated with 12 EST-SSRs. Maps: colored pie charts show the total ratios of assigned genetic clusters of individuals in each population when K = 3 (**A**) and when K = 4 (**B**). (**C**) Results were plotted for K = 3 and K = 4 based on the most suitable, average log-likelihood of the data at K plus/minus its standard deviation across runs (meanL(K) ± SD), mean estimated log probability of the data (mean Ln P(D)), and delta K (ΔK) value. The graphical output of the assigned genetic cluster is represented with the color scheme.

The genetic structure of the 13 studied *H. italicum* populations was also assessed using Bayesian clustering in STRUCTURE. At K = 3 (meanL(K) ± SD = 3, mean Ln P(D) = −7366.36, ΔK = 51.716), Ajaccio formed a distinct western cluster, and Capo Pertusato, Pianottoli, and Tonnara grouped as a southern cluster, while all remaining populations were assigned to a single cluster. At K = 4 (meanL(K) ± SD = 4, mean Ln P(D) = −7319.48, ΔK = 13.14), a finer resolution revealed geographically coherent clusters: Ajaccio remained separate; the southern populations of Capo Pertusato, Pianottoli, and Tonnara formed a distinct group; and Plage de l’Ovu Santu, Conca, Punta di a Vacca Morta, Col de Bavella, and Col de Saint-Eustache formed the southeastern cluster. Partial admixture was observed in Corte, Sagone, Piana, and the Croatian population Cape Kamenjak (Figure 6).

After the clustering results from STRUCTURE (K = 4) were obtained, basic population genetic parameters were recalculated for each of the four genetic clusters (Table 9). In all genetic clusters, the observed heterozygosity was higher than the expected heterozygosity (H_o_ = 0.583–0.684; H_e_ = 0.492–0.576). Genetic cluster 4 (Corte, Sagone, Piana, and Cape Kamenjak) exhibited the highest number of private alleles (seven), followed by genetic cluster 2 (Capo Pertusato, Pianottoli, and Tonnara) with four, genetic cluster 3 (Plage de l’Ovu Santu, Conca, Punta di a Vacca Morta, Col de Bavella, and Col de Saint-Eustache) with three, and genetic cluster 1 (Ajaccio) with one private allele. Allelic richness was highest in genetic cluster 3 (3.560) and lowest in genetic cluster 1 (3.010), whereas private allelic richness was highest in genetic cluster 4 (0.420).

## 3. Discussion

Medicinal and aromatic plants (MAPs) are globally recognized as an important source of bioactive compounds, with individual plant parts (roots, leaves, stems, etc.) or whole plants widely used across industries [75]. Since most MAPs are non-model higher plant species, genomic and transcriptomic resources are still scarce despite many years of high-throughput sequencing technologies [76,77]. However, rapid development of next-generation sequencing has contributed to an increasing number of genomic and transcriptomic studies [78,79], opening additional possibilities for the identification of genes involved in secondary metabolite synthesis and the development of markers for diversity studies and plant breeding. EST-SSRs, which can be developed from de novo transcriptomes [80], are valuable molecular markers for assessing genetic variation and supporting marker-assisted selection, as they are directly linked to expressed genes. However, despite the importance of *H. italicum*, genomic and transcriptomic studies on this species remain limited.

In this paper, the first de novo transcriptome assembly of *H. italicum* is reported. The selected assembly (rnaSPAdes assembly with k-mers 21, 33, 55, 77, 99, and 127) comprised 24,806 contigs with an N50 of 2030 bp. This contig N50 was longer than those reported for other non-model MAP species in the Asteraceae family, such as *Saussurea amara* (N50 = 1562 bp; [81]), *Achillea acuminata* (N50 = 1678 bp; [82]), and *Celmisia lyallii* (N50 = 931 bp, [83]). It should be mentioned that larger number of transcripts were assembled in those studies (185,952 for *S. amara*; 177,816 for *A. acuminata*; and 223,036 for *C. lyallii*). In addition to the N50, the high proportion of reads mapping back to the assembly (93.82%) confirmed the quality of the assembly, comparable to that reported in the work by Tribhuvan et al. [84] on *Artocarpus heterophyllus* (Moraceae). In total, 84.8% of the assembled transcripts were assigned to the Asteraceae family or to a lower taxonomic level such as *Erigeron canadensis* (14.9%), *Cynara cardunculus* (5.5%), *Helianthus annuus* (3.6%), and *Artemisia annua* (2.5%). The species distribution was comparable to that reported for *Aster spathulifolius*, where top-hit species similarity was highest with *C. cardunculus* (24.41%), *A. annua* (19.20%), and *H. annuus* (1.3%) [85].

In total, 55.4%, 62.5%, and 68.3% of transcripts were assigned to KEGG orthology, GO terms, and KOG functional groups, respectively. Significant hits on protein databases—UniProtKB/Swiss-Prot, RefSeq Protein, and NCBI nr—were obtained for 89% to 99.5% transcripts. Transcripts annotated to KOG category ‘Secondary metabolites biosynthesis, transport. and catabolism’, KEGG pathways involved in secondary metabolite biosynthesis and plant–pathogen interaction, or GO terms related to biosynthesis of secondary metabolites and response to abiotic and biotic factors could be prioritized in further studies to assess their functions in adaptation to environmental conditions and industry-relevant traits. Functional annotation was also considered for the selection of EST-SSR loci.

In plant sciences, EST-SSRs have been successfully applied for the assessment of genetic diversity [86], population structure analysis [87], marker-assisted selection [88], and construction of genetic linkage maps [89]. EST-SSRs thus provide a molecular tool and a potential link to a gene function, which also contributes to the understanding of biosynthetic pathways in plants [90]. Since only two sets of microsatellite markers have been developed specifically for *H. italicum* [48,49], the development of EST-SSR markers appears to be pertinent to the gathering of complementary genetic information.

To minimize redundancy in microsatellite detection, transcripts from the de novo assembled transcriptome were clustered into 19,921 unigenes, across which 2017 microsatellite loci were detected (excluding mononucleotide and compound microsatellites). Among them, the trinucleotide repeats were the most abundant, followed by dinucleotides. These results are consistent with the development of EST-SSRs in other medicinal and aromatic plants, such as *Epimedium sagittatum* [91], *Pseudostellaria heterophylla* [92], and *Saussurea costus* [93]. Among the dinucleotide repeat motifs, TA/TA (24.7%) and AT/AT (24.5%) were the most common. Similarly, Xu et al. [92] found the AT/AT repeat to be the most common in *Pseudostellaria heterophylla*. Among the trinucleotide repeat motifs, ATC/GAT (13.7%) and TGA/TCA (10.7%) were the most common. The same two motifs were also reported as the most common in *Chrysanthemum* × *morifolium* [94]. In contrast, the TGA motif (which represents a stop codon in an open reading frame) was not among the most common trinucleotide motifs in several other studies—for example Scaglione et al. [95] found the AAG/CTT, ATC/GAT and CAC/GTG motifs as the most common in *Cynara cardunculus* var. *scolymus*, while Li et al. [96] found the AAT/ATT, AAG/CTT, and ATC/GAT repeats to be the most common in *Zanthoxylum bungeanum*.

After the initial amplification of 78 EST-SSR loci, 30 (38.5%) produced clear and scorable fragments, of which 23 loci (76.7%) were polymorphic. Considering the limited number of microsatellite markers previously available for *Helichrysum* species, we also investigated the possibility of transferability of the newly developed EST-SSRs to other *Helichrysum* species. All 23 polymorphic EST-SSRs were successfully amplified in *H. arenarium* and *H. litoreum*, thereby contributing to a larger number of available microsatellite markers for genetic studies. The cross-transferability of new EST-SSRs is of particular importance for *H. arenarium* as natural populations are threatened, and genetic resources remain scarce [50]. It is legally protected in countries such as Sweden and Serbia, while in Denmark and Estonia, it is classified as ‘care demanding’ [97]. In addition, the flowers of *H. arenarium* are recognized in several national pharmacopoeias, including the Russian, Swiss, and Polish ones, for their cholagogue and choleretic activities [97]. These facts emphasize the value of reliable EST-SSRs for both conservation strategies and the sustainable utilization of this medicinally important species [96]. Establishing *H. litoreum* cross-transferability appears to be important since this species grows together with *H. italicum* in parts of their natural range (west–central Italy, several Tyrrhenian islands, and the Istrian peninsula). These plants can hybridize, and plants with intermediate morphology are often found, making identification based solely on external traits unreliable [98,99]. In this context, such molecular markers are essential for their differentiation as well as for further taxonomic and population genetic studies.

For further characterization, only 12 EST-SSRs with at least three alleles were retained and used on a large sample (270 individuals from 13 natural populations) of *H. italicum*. According to the polymorphic information content (PIC), six EST-SSRs were regarded as highly informative (PIC > 0.5) and six EST-SSRs as reasonably informative (0.5 > PIC > 0.25), while no EST-SSRs had a PIC < 0.25 and were thus considered as only slightly informative [100]. Compared to genomic SSRs, with a mean PIC value of 0.707 [48], the new EST-SSRs in *H. italicum* were less polymorphic, with a lower mean PIC value of 0.571. Since DNA sequences tend to be more conserved in transcribed regions [90], lower polymorphism is expected for those genomic regions. Such results were observed also in other studies [101,102]. The Hardy–Weinberg equilibrium (HWE) is often used as a starting point for all population genetics investigations [103]. In this study, the HWE analysis of the 12 EST-SSR markers revealed that seven EST-SSRs showed no significant deviation from HWE, while five EST-SSRs deviated significantly (*p* < 0.05). For loci EST-HiUP-01 and EST-HiUP-21, the observed result could be due to different allele frequencies in different populations. In such cases markers can show deviation from HWE, and such deviations can be misinterpreted as a potential problem in genotyping quality, resulting in false exclusion from future analysis [104]. In contrast, loci EST-HiUP-02, EST-HiUP-13, and EST-HiUP-14 showed high heterozygosity, which could result from several factors, including the self-incompatibility mechanism characteristic of Asteraceae [105] and are therefore expected to deviate from HWE. The presence of null alleles was also assessed, but none were detected in any of the 12 EST-SSRs, as all estimated values of F_(null)_ were below the threshold (*p* < 0.2) that would indicate the presence of ‘true’ null alleles [106].

To evaluate the genetic diversity of 12 populations of *H. italicum* in Corsica, we used calculations for multiple genetic parameters. The observed heterozygosity (H_o_ = 0.567–0.742) was higher than expected (H_e_ = 0.477–0.598) in all populations, which is characteristic for cross-pollinated species. *H. italicum* is an insect- and wind-pollinated plant [46], and such pollination promotes allele exchange among individuals, maintaining high levels of heterozygosity and genetic diversity within populations [107]. Shannon’s information index (I) was used to describe genetic variation based on EST-SSRs at the species level. Population Col de Saint-Eustache had the highest genetic variation detected (I = 1.129), while Pianottoli had the lowest (I = 0.878). Private alleles were detected in Corsican populations of Plage de l’Ovu Santu (three alleles), Piana (two alleles), Tonnara (two alleles), Conca (one allele), and Col de Saint-Eustache (one allele). These alleles may help describe differences among populations, but as they occurred at low frequencies, they cannot be reliably linked to taxonomic differences without additional evidence.

Interestingly, despite the populations of *H. italicum* occurring in the island ecosystems of Corsica—an area with known glacial refugia [108]—being geographically isolated and affected by human activities (e.g., urbanization, and the collection of plants from natural populations), no bottleneck event has yet occurred in the analyzed natural populations, as the probability values were above 0.05. However, this does not exclude the possibility that bottleneck events may have occurred in the past and that populations subsequently recovered, leaving no detectable genetic signal in the present data. The pairwise F_st_ analysis revealed the highest differentiation between the populations of Corte and Pianottoli, with the F_st_ value of 0.067. This F_st_ value is lower than those reported in other insular Asteraceae populations, i.e., *Centaurea filiformis*, with F_st_ = 0.099 and overall F_st_ = 0.218 [109]. Accordingly, the AMOVA revealed that there is more genetic variance among individuals within populations (85%) than there is among populations (15%), suggesting a high level of intra-population genetic diversity and the low geographical structuration observed. A completely comparable pattern was found by Ninčević et al. [39], who studied population structure of 18 wild *H. italicum* populations sampled along the eastern Adriatic coast with AFLP markers. Their results also showed higher intrapopulation diversity than interpopulation genetic diversity, indicating extensive gene flow between *H. italicum* populations.

To evaluate the population structure, we used DAPC, which does not rely on a particular population genetics model [110] and the Bayesian clustering method implemented in STRUCTURE to reveal additional patterns of clustering. Both approaches revealed the distinctness of the Ajaccio population. The Ajaccio population represents the westernmost sampling site, located in an open, wind-exposed habitat, named La Parata. The presence of *H. italicum* subsp. *italicum* near Ajaccio was previously documented in botanical surveys [111,112]. However, it is noteworthy that La Parata was reported as the only western locality where *H. italicum* subsp. *tyrrhenicum* (formerly subsp. *microphyllum*) was found (Jeanmonod [113], as reviewed in Galbany-Casals et al. [46]). Otherwise, the distribution area of *H. italicum* subsp. *tyrrhenicum* is primarily restricted to the southern coast [45]. Therefore, the Ajaccio population could represent a unique genetic structure, potentially shaped by stronger selection pressure in response to environmental conditions. La Parata is a granitic cone with a high percentage of halophiles species in its vegetation [114]. Additionally, the potential occurrence of *H. italicum* subsp. *tyrrhenicum* in the areas surrounding Ajaccio may also contribute to the observed population. However, distinguishing between *H. italicum* subsp. *italicum* and *H. italicum* subsp. *tyrrhenicum* in areas where their distribution overlaps remains challenging [45], as gene flow can blur the morphological and genetic boundaries between them. Populations from Capo Pertusato, Pianottoli, and Tonnara, located within the core distribution area of *H. italicum* subsp. *tyrrhenicum* [45], formed a single cluster in STRUCTURE but were divided into two clusters by DAPC (Capo Pertusato samples were mostly assigned to cluster 2, whereas the southwestern sites—Pianottoli and Tonnara—were grouped in cluster 4 with several individuals assigned in cluster 2). It is possible that DAPC detected differences in genetic structure driven by environmental factors. Capo Pertusato and the Tonnara/Pianottoli area differ in their ecological profiles, although they share the general exposure to coastal conditions. Capo Pertusato is situated on Miocene limestone overlain by sand [115], whereas Tonnara beach is interspersed with granitic blocks [114]. However, in all analyses, the southern Corsican cluster, likely corresponding to *H. italicum* subsp. *tyrrhenicum*, appeared to be (significantly) different to the Ajaccio population. STRUCTURE analysis with three (K = 3) and four (K = 4) genetic populations divided all other populations into two main groups corresponding to geographic origin, separating central–western/northern Corsican populations from those in the south of the island. Since all populations, except Sagone and Plage de l’Ovu Santu, are located inland, where only *H. italicum* subsp. *italicum* occurs [46], they belong to this subspecies. As in the previous case, the DAPC further subdivided the samples of these populations, and clustering patterns revealed similarities between certain populations such as Conca and Col de Saint-Eustache. It is noteworthy that the population of Punta di a Vacca Morta, geographically closest to the distribution area of *H. italicum* subsp. *tyrrhenicum*, was subdivided into seven of the eight DAPC clusters, a pattern likely reflecting ongoing gene flow among neighboring populations. Although the population from Kamenjak (Croatia) was included as an outgroup, it was not genetically completely separated from the Corsican populations. These observations are consistent with the findings of Galbany-Casals et al. [46], who divided *Helichrysum italicum* into a western (Spain, France, Italy, Croatia) and an eastern genetic group (Greece, Cyprus) and found high chloroplast DNA polymorphism within the western Mediterranean group, both between and within populations.

The EST-SSRs revealed a clear genetic pattern distinguishing southernmost and northern *H. italicum* populations. Consistent with previously reported geographical gradients in morphological traits [46,98] and the fact that *H. italicum* subsp. *tyrrhenicum* is restricted to the southern part of Corsica [45], our genetic population analysis showed a similar trend and coincided with taxonomic classification. This suggests that the developed EST-SSRs are able to differentiate *H. italicum* subsp. *tyrrhenicum* from *H. italicum* subsp. *italicum*.

## 4. Materials and Methods

### 4.1. Plant Material

**Figure 7:**
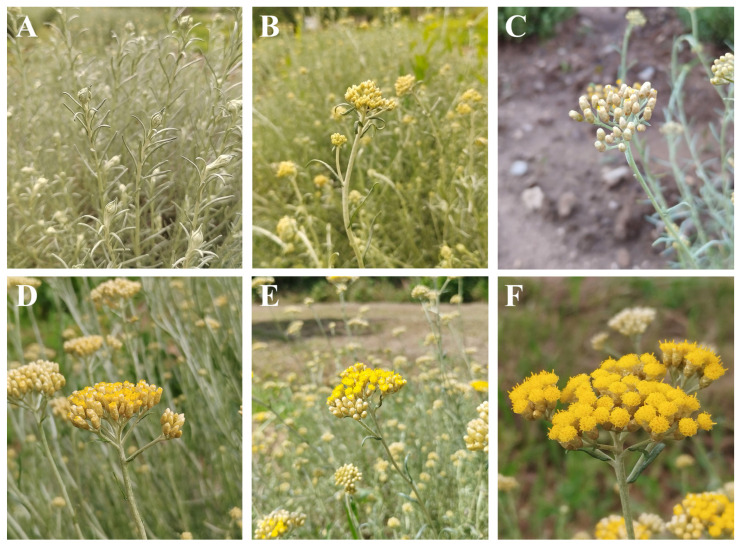
Phenological stages of the six sampling points during one month of *H. italicum* vegetative growth. (**A**) Inflorescence emergence. (**B**) First individual flowers. (**C**) First flower petals. (**D**) Beginning of flowering. (**E**) Full flowering. (**F**) End of flowering.

For the transcriptome sequencing young shoots with leaves and flower buds were collected from one-year-old and disease-free plants of *H. italicum* subsp. *italicum* grown at the ex situ collection of the University of Primorska, Faculty of Mathematics, Natural Sciences and Information Technologies, in Ankaran, Slovenia (45°34′19.3″ N 13°46′33.2″ E). *H. italicum* shoots were sampled during one month of vegetative growth, from the end of May until the end of July 2019, including two principal growth stages, inflorescence emergence and flowering [116]. Sampling was carried out once per week, thus yielding six sampling time points and consequently six distinct phenological stages (Figure 7). To ensure an adequately representative sample, each sample consisted of four young shoots from four individual *H. italicum* shrubs with identical genetic profiles. Each bulk sample was immediately snap frozen in liquid nitrogen, ground to powder with a pestle in a mortar, and stored at −80 °C in the genetic laboratory of UP FAMNIT until further use.

**Figure 8:**
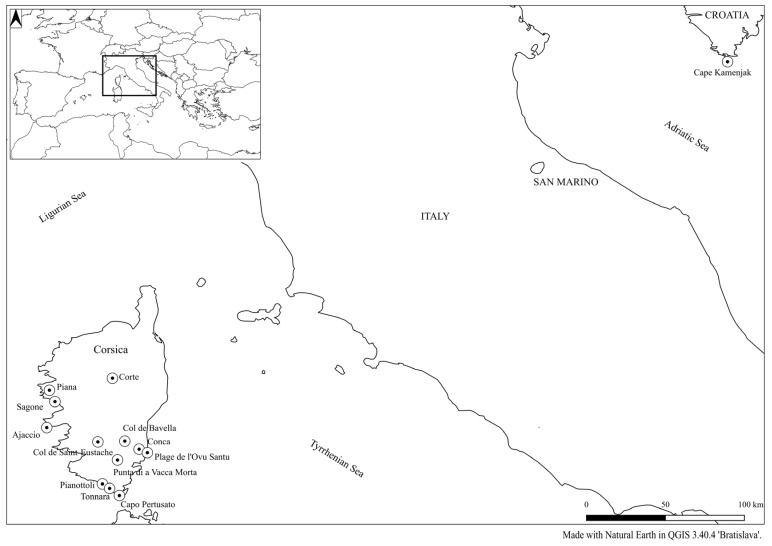
Collection sites of *H. italicum* in France (Corsica) and Croatia (Istrian peninsula).

Plants that were at least 10 m apart were sampled in populations of *H. italicum* in 12 locations across Corsica and in 1 location in the southernmost cape of the Istrian peninsula (Figure 8). The young shoots were immediately stored in 2 mL tubes with the saturated and highly viscous NaCl-CTAB solution [117] to prevent DNA degradation and plant material oxidation. Samples were stored at 2–8 °C in the genetic laboratory of UP FAMNIT until further use. Commercially available *H. arenarium* (from dried herbal tea, purchased from Flora Ltd., Rogatec, Slovenia) and samples of *H. litoreum* were included for a cross-species transferability test (Table 10). The collection of plant material was conducted in accordance with the Nagoya Protocol and was carried out under a declaration issued by the French Ministry for Ecological and Solidary Transition (Ministère de la Transition écologique et de la Cohésion des territoires) (NOR TREL 2002508 S/338). Sampling in the Istrian peninsula (Croatia) was carried out under an agreement of cooperation with the Istrian Botanical Society.

### 4.2. RNA Extraction and cDNA Library Preparation for Transcriptomic Sequencing

The Spectrum™ Plant Total RNA kit (Sigma-Aldrich, St. Louis, MO, USA) was used for extracting total RNA from approximately 100 mg of frozen plant material following the manufacturer’s protocol. The quantity and quality of six RNA samples (each belonging to one sampling date) was assessed using an Epoch spectrophotometer (BioTek, Winooski, VT, USA) and with the Agilent RNA 6000 Nano Kit on the Agilent 2100 Bioanalyzer (Agilent technologies, Waldbronn, Germany). Based on measurements equal amounts of each RNA sample were combined, and finally, the mRNA of pooled RNA samples was enriched with Dynabeads™ mRNA DIRECT™ Micro Purification kit (Thermo Fisher Scientific, Waltham, MA, USA).

The high-quality enriched mRNA was processed further for cDNA library preparation. The cDNA library was prepared with Ion Total RNA-Seq Kit v2 (Thermo Fisher Scientific, Vilnius, Lithuania). Next, the cDNA library concentration was evaluated using Qubit™ dsDNA HS Assay Kit (Thermo Fisher Scientific, Eugene, OR, USA) and Qubit™ 3.0 fluorometer (Thermo Fisher Scientific, Kuala Lumpur, Malaysia), and the library quality was assessed with the High Sensitivity DNA kit on an Agilent 2100 Bioanalyzer (Agilent technologies, Waldbronn, Germany). The cDNA library was diluted to 100 pM according to Qubit and Bioanalyzer measurements. The library was amplified on an Ion OneTouch™ 2, and template positive Ion Sphere Particles were enriched with the Ion OneTouch™ ES System (Thermo Fisher Scientific, Carlsbad, CA, USA) using Ion 520™ & 530™ OT2 Kit (400 bp option) (Thermo Fisher Scientific, Waltham, MA, USA). Finally, the enriched library was loaded on the Ion 530™ chip (Thermo Fisher Scientific, Waltham, MA, USA) and sequenced on an Ion GeneStudio™ S5 System (Thermo Fisher Scientific, Marsiling Industrial Estate, Singapore).

### 4.3. De Novo Transcriptome Assembly and Data Analysis

The raw sequencing data generated by the Ion GeneStudio™ S5 System (Thermo Fisher Scientific, Marsiling Industrial Estate, Singapore) were first processed through FastQC tool version 0.11.9 [118] for quality control check and to spot the presence of ion torrent adaptors at the first positions of the reads. The de novo transcriptome assembly was performed using two different software packages. (1) The first was rnaSPAdes software version 3.15.4 [119] with iontorrent flag (required for IonTorrent data which allows bam file as input) and the strand specificity set to forward (fr), and two different k-mer length settings were tested: -k 21, 33, 55, and 77; and -k 21, 33, 55, 77, 99, and 127 (hereby referred to as ‘rnaSPAdes’ (77) and ‘rnaSPAdes’ (127), respectively). (2) The second was MIRA software version 5rc1 [120,121], with mirabait being used to clean out ribosomal RNA sequences from the data set and the following command line being used for assembly: job = denovo, est, accurate. The quality of all assemblies was assessed by examination of the orthologues completeness using BUSCO version 5.3.2 [122] and eudicots_odb10 database with 2326 genes, and the summary statistic of assembled transcripts was obtained with rnaQUAST version 2.2.0 [123].

In the next step, the quality of rnaSPAdes assembly was further assessed by mapping raw reads back to the assembled contigs using TMAP (https://github.com/iontorrent/TS/tree/master/Analysis/TMAP, accessed on 10 May 2023) version 3.4.0. Finally, SALMON version 1.5.2 [124] was used for gene expression level estimation. The raw sequences generated are available from the NCBI Short Read Archive (SRA) under the accession number PRJNA1308477.

### 4.4. Functional Annotation

Functional annotation of assembled transcripts was first done using the clustering module of MMseqs2 version 13.45111 software [125,126] (mmseqs search workflow), and the contigs were aligned against the NCBI Nr database and NCBI RefSeq_Protein database (https://www.ncbi.nlm.nih.gov/refseq/; accessed on 25 April 2022). Similarly, MMseqs2 was used for taxonomical classification (mmseqs taxonomy workflow) based on the NCBI Nr database to detect possible contamination with transcripts of non-plant organisms.

Further functional annotation of assembled transcripts was done using EggNOG software version 2.1.7 (https://github.com/eggnogdb/eggnog-mapper; accessed on 15 June 2022) to obtain annotations from COG (https://www.ncbi.nlm.nih.gov/COG/; accessed on 15 June 2022; clusters of orthologous groups), KOG, and GO (http://geneontology.org; accessed on 15 June 2022; Gene Ontology). GO annotations were subjected to GOATOOLS [127] to map GO terms to GO plant slims using map2slim.py script and wr_hier.py script for hierarchical grouping of GO slims. The assembled transcripts were also compared with UniProtKB/Swiss-Prot database using BLAST+ version 2.15.0 (BlastX). Finally, transcripts were subjected to KAAS server (http://www.genome.jp/kegg/kaas/; accessed on 15 June 2022; KEGG Automatic Annotation Server) to obtain KEGG Orthology annotations, and the reconstruct tool was used to map KO identifiers to the KEGG pathway maps.

### 4.5. Identification of Microsatellite Repeats (SSRs) and Primer Development

The clustering analysis (mmseqs cluster workflow) of selected transcriptome assembly was performed using MMseqs2 to acquire non-redundant transcripts (unigenes). All unigenes obtained with MMseqs2 (mmseqs cluster workflow) were analyzed with MIcroSAtellite Identification Tool (MISA) version 2.1 (http://pgrc.ipk-gatersleben.de/misa/; accessed on 12 October 2022). The parameters were set as follows: a minimum of six repeat units for dinucleotide and five repeat units for tri, tetra, penta, and hexanucleotide motifs. Notably, mononucleotide and compound microsatellites were not included in this study.

TMAP alignment was examined for annotated SSR-containing unigenes with assembly viewer Tablet [128] to select microsatellites flanked by appropriate regions for primer design with Primer3 version 2.6.1, installed via the Bioconda channel [129,130]. Results from MISA were piped into Primer3 using a modified PERL script (available at https://github.com/soltislab/transcriptome_microsats.git, accessed on 12 October 2022).

For further selection of suitable transcripts with found microsatellite motifs, the intron presence between developed primer sites was assessed based on multiple sequence alignment (MSA), including unigene, mRNA of RefSeq protein obtained previously with MMSeqs2, and the corresponding genome sequence. The MSA for each unigene was produced with MAFFT software version 7.526 [131]. Sequence alignments were manually assessed, and only loci with both primer sites on the same exon and showing an almost perfect alignment were selected. Location of the SSR (UTR or CDS region) was determined based on RefSeq mRNA annotations. Furthermore, protein and KEGG annotations were examined to focus on biosynthetic pathways for the metabolism of terpenoids and polyketides or biosynthesis of other secondary metabolites. Additional information was obtained with a manual search in scientific publications.

### 4.6. EST-SSR Preliminary Amplification and Cross-Species Transferability

The extraction of genomic DNA from plant tissue was done according to the adjusted CTAB-PVP protocol [48,132]. The precipitated DNA was dissolved in TE (Tris-EDTA) buffer and stored at −20 °C. The DNA concentration was quantified using Qubit™ dsDNA BR Assay Kit (Thermo Fisher Scientific, Eugene, OR, USA) and Qubit^TM^ 3.0 fluorometer (Thermo Fisher Scientific, Kuala Lumpur, Malaysia), and the DNA was diluted to 10 ng/µL. The isolated DNA samples were deposited in the genetic laboratory of UP FAMNIT under the accession numbers HIUP_1-270 (*H. italicum*), HAUP_1-6 (*H. arenarium*), and HLUP_1-6 (*H. litoreum*).

The 78 developed EST-SSR primers were first tested on eight *H. italicum* samples according to the economically fluorescent-dye method by Schuelke [133]. PCR reaction was performed in a final volume of 12.5 µL containing 40 ng DNA, 1× supplied AllTaq PCR buffer, 1× supplied Q-Solution, 2 mM of MgCl_2_, dNTP mix (0.2 mM of each dNTP), 1.25 U of AllTaq DNA polymerase (Qiagen, Hilden, Germany), 0.2 mM of reverse locus specific primer and M13(-21) tail labeled with fluorochrome (6-FAM, VIC, NED, or PET), and 0.1 mM of elongated forward primer. Locus-specific primers were synthesized by IDT-DNA and universal M13(-21) primer by Applied Biosystems (Thermo Fisher Scientific, Foster City, CA, USA).

The conditions of the two-step PCR consisted of an initial denaturation at 94 °C for 5 min, followed by 35 cycles of denaturation for 30 s at 94 °C and for 45 s at the annealing temperature of 56 °C, and the extension at 72 °C for 45 s. The second step of amplification passed through eight cycles of 30 s at 94 °C and at 45 s at the annealing temperature of 53 °C and 45 s of elongation at 72 °C, followed by a final extension for 8 min at 72 °C. Additionally for loci where amplification presented multiple alleles, the annealing temperature was experimentally optimized to 58 °C, 60 °C, 62 °C, and 64 °C (Table 5). PCR products were prepared with Hi-Di^TM^ Formamide (Thermo Fisher Scientific, Woolston, UK) and GeneScan^TM^ 500 LIZ (Thermo Fisher Scientific, Woolston, WA, UK) as a size standard for the fragment analysis with capillary electrophoresis on a SeqStudio^TM^ Genetic Analyzer (Thermo Fischer Scientific, Marsiling Industrial Estate, Singapore). Scoring of the electropherograms was done in GeneMapper version 5 computer software (Thermo Fisher Scientific, Waltham, MA, USA).

Twenty-three finally selected polymorphic EST-SSRs (Table 5) were tested for their potential transferability to closely related *Helichrysum* species, namely *H. litoreum* and *H. arenarium*, using the same PCR protocol as that used for the *H. italicum* samples. Sequences with primers were deposited in the NCBI Nucleotide database with accession numbers from PX219824 to PX219846.

### 4.7. Characterization of EST-SSR Markers and Population Analysis of H. italicum

A set of 12 polymorphic EST-SSRs, with at least three alleles obtained at the preliminary amplification, was used to study the genetic diversity of 12 populations of *H. italicum* from Corsica (France). As an outgroup, one population from the Istrian peninsula (Croatia) was included. The number of alleles (N_a_), the number of effective alleles (N_e_), the expected heterozygosity (H_e_), the observed heterozygosity (H_o_), and the Shannon’s information index (I) were calculated for 12 EST-SSRs on 270 samples of *H. italicum* using the computer software GenAlEx version 6.503 (Research School of Biology, The Australian National University, Acton, Australia) [134]. All microsatellites were tested for the presence of stuttering, null alleles, and large allele dropout using Micro-Checker version 2.2.3 software [135]. The polymorphic information content (PIC), Hardy–Weinberg equilibrium (HWE), and the frequency of null alleles (F_null_) were calculated with the program Cervus version 3.0.7 [136].

To assess the genetic variability within *H. italicum* populations, the N_a_, N_e_, H_e_, H_o_, I, inbreeding coefficient (F), and potential private alleles were calculated using GenAlEx. To test for recent population bottlenecks, the Wilcoxon signed-rank test [137] for heterozygosity excess was applied under the two-phase model (TPM) with 10,000 iterations and 95% single-step mutations (SSMs) via the program BOTTLENECK version 1.2.02 [138].

The genetic variability between *H. italicum* populations was assessed using several complementary approaches. Analysis of molecular variance (AMOVA) and the estimation of the proportion of total genetic differentiation (F_st—_Fixation index) were performed using GenAlEx. Discriminant analysis of principal components (DAPC) was conducted with the R package ‘adegenet’ version 2.1.11 [139] to identify and describe clusters from 270 individuals belonging to 13 *H. italicum* populations. K-means analysis (in the function ‘find.clusters’) of principal-component (PC)-transformed genotyping data and the Bayesian information criterion (BIC) were used to determine the optimal number of clusters. For the DAPC analysis, the number of clusters (K) was the one at the ‘elbow’ point of the BIC vs. K plot and that had the lowest BIC value. With the ‘xvalDapc’ we performed the cross-validation of DAPC using varying numbers of PCs to determine the optimal number of PCs to retain. For visualization, we used a scatterplot of the DAPC and a heatmap of inferred DAPC clusters [110,140]. The Bayesian clustering method implemented in STRUCTURE version 2.3.4 [141] was used to estimate the number of clusters (K) based on EST-SSR data. The parameters used in the analysis were the length of Burnin period 100 k, number of MCMC reps after Burnin 100 k, a K set from 1 K to 15 K, and five iterations. STRUCTURE results were analyzed with the R package ‘pophelper’ version 2.3.1 [142] to infer the most likely number of genetic clusters using the Evanno method [143]. The output matrix for the best K-value according to the Evanno method (K = 3) and geographically coherent clustering (K = 4) were plotted with the R package ‘pophelperShiny’ version 2.1.1 [144]. For the set of genetic groups (K = 4) inferred by STRUCTURE, the H_o_, H_e_, and the potential private alleles were calculated using GenAlEx, while allelic richness (N_ar_) and private allele richness (N_par_) were estimated with HP-RARE version 1.0 [89].

## 5. Conclusions

This paper presents the first de novo transcriptome assembly of *Helichrysum italicum* subsp. *italicum*, providing a valuable genomic resource for this non-model but highly important medicinal and aromatic plant. The newly developed EST-SSRs considerably expand the number of available microsatellite markers, not only for *H. italicum* but also for other species of the genus *Helichrysum*. Combining molecular markers with chemotype profiles is essential for careful selection of plant material with desirable characteristics for cultivation. These markers represent a powerful tool for authentication and traceability, which is essential to preventing adulteration in the herbal industry as well as fraud and mislabeling in the market. Furthermore, such markers are of great importance for the development of quality certifications such as protected geographical indication (PGI) for *H. italicum* from a given geographical area and for the development of new cultivars through future breeding programs. Our study revealed geographically coherent patterns, indicating some degree of local adaptation. These findings can guide the selection of representative germplasm from highly diverse natural populations and support the identification of potentially valuable genotypes.

Results of the population analysis represent an important step towards the assessment of genetic diversity and population structure in the geographically distinct Corsican populations of *H. italicum* and towards the adjustment of conservation strategies for this morphologically and genetically diverse Mediterranean plant.

Together, these achievements form the foundation for future breeding, domestication, and conservation efforts in *H. italicum*, ensuring that both economic interests and long-term genetic resources are maintained.

## Figures and Tables

**Table 1:** Summary statistics of de novo assemblies and completeness evaluation with BUSCO based on eudicots lineage (*n* = 2326 genes). rnaSPAdes (77) refers to the assembly using k-mer sizes of 21, 33, 55, and 77, whereas rnaSPAdes (127) refers to the assembly using k-mer sizes of 21, 33, 55, 77, 99, and 127; and the MIRA assembly.

Category	rnaSPAdes (77)	rnaSPAades (127)	MIRA
Total assembled transcripts	43,359	24,806	233,132
Assembled transcripts > 1000 bp	33,383	21,284	9340
Average length of assembled transcripts (bp)	1498	1832	273
N50 (bp)	1572	2030	358
Complete BUSCOs (%)	49.2	48.6	43.6

**Table 2:** Taxonomical assignment of *H. italicum* assembled transcripts against NCBI Nr (nd—not defined).

Taxonomical Classification							Proportion (%)
Kingdom	Phylum	Class	Order	Family	Genus	Species	
Viridiplantae	Streptophyta	Magnoliopsida	Asterales	Asteraceae	*Erigeron*	*E. canadensis*	14.9
					*Cynara*	*C. cardunculus*	5.5
					*Helianthus*	*H. annuus*	3.6
					*Artemisia*	*A. annua*	2.5
					*Tanacetum*	*T. cinerariifolium*	1.1
					*Mikania*	*M. micrantha*	1.0
					*Lactuca*	nd	1.8
					nd	nd	54.4
			nd	nd	nd	nd	11.3
		nd	nd	nd	nd	nd	1.3
nd	nd	nd	nd	nd	nd	nd	2.5

**Table 3:** Summary of the different de novo transcriptome annotations.

Database	Number/Proportion (%) of Annotated Transcripts (*n* = 24,738)
NCBI Nr annotated	24,621/99.5
RefSeq Protein	24,588/99.1
UniProtKB/Swiss-Prot	22,036/89.0
EggNOG	24,395/98.6
COG (EggNOG database)	12,441/50.0
KOG (EggNOG database)	16,894/68.3
GO (EggNOG database)	15,453/62.5
KEGG Orthology (KO)	13,711/55.4

**Table 4:** Summary of the microsatellite repeat identification in *H. italicum* unigenes (excluding mononucleotide and compound microsatellites).

Item	Number
Total number of examined unigenes	19,921
Total size of examined unigenes (bp)	34,836,960
Total number of identified SSRs	2107
Number of SSR-containing unigenes	1958

**Table 5:** Characteristics of the 23 polymorphic EST-SSRs.

GenBank Accession Number	Locus	Primer Sequence (5′-3′) *	Repeat Motif	SSR Location	Size Range (bp)	Ta (°C)	Putative Function; Accession Code
PX219824	EST-HiUP-01	F: CGTTGAACACGGAGGTAGTGA R: ACCGTGTATGCATGCTCCAA	(TGA)_6_	CDS	204–213	56, 60 **	ABC transporter B family member 2-like [*Cynara cardunculus* var. *scolymus*]; XP_024993859.1
PX219825	EST-HiUP-02	F: TTCCGGATATGGTGGTGCTG R: CCAACAGCACCATACCCAGT	(TGG)_5_	CDS	204–210	56, 60 **	heterogeneous nuclear ribonucleoprotein 1 [*Cynara cardunculus* var. *scolymus*]; XP_024984907.1
PX219826	EST-HiUP-03	F: AGAATTGTGGCGGATGACGT R: AGGGGGCAAAACAGAAGTGT	(GGT)_6_	CDS	262–279	56, 58 **	mediator of RNA polymerase II transcription subunit 14 [*Erigeron canadensis*]; XP_043627804.1
PX219827	EST-HiUP-04	F: TGGGATTGGGATTAATTGGCGA R: ATCGAGGGGTCGGTTATGGT	(CAA)_5_	CDS	187–190	56	protein TWIN LOV 1 [*Cynara cardunculus* var. *scolymus*]; XP_024989500.1
PX219828	EST-HiUP-05	F: CGGACGCGGTAGAACATGAT R: CGCAACTGTAAGGCCTCTGA	(ACCAAT)_5_	CDS	117–142	56	heparan-alpha-glucosaminide N-acetyltransferase [*Lactuca sativa*]; XP_023765509.1
PX219829	EST-HiUP-06	F: CACTCCATTTGTGATGTCAACCA R: TGAAACCGGGAAGAAGCGAA	(CGC)_5_	CDS	168–177	56	3-deoxy-manno-octulosonate cytidylyltransferase, mitochondrial [*Erigeron canadensis*]; XP_043632357.1
PX219830	EST-HiUP-07	F: GGCGAGTACTCCGTACAACC R: ACAGTAATGGAAGCCAAACAACT	(AT)_6_	3′ UTR	246–252	56	serine acetyltransferase 2-like [*Erigeron canadensis*]; XP_043632354.1
PX219831	EST-HiUP-08	F: ACCAATCAGGATTTGCGGGT R: GGCTGCCGTGAAGTTAGGAT	(CAC)_5_	CDS	177–180	56	WRKY transcription factor 6 [*Helianthus annuus*]; XP_022036883.1
PX219832	EST-HiUP-09	F: CGACATCCCGTGTATCCCAG R: TGCTCCTTTGACAGAAACCCA	(AT)_11_	3′ UTR	249–251	56	GDSL esterase/lipase At5g55050 [*Erigeron canadensis*]; XP_043606389.1
PX219833	EST-HiUP-10	F: CAAACGGCACCATTTCAGCA R: TGGGCCGGATAGAAAAACCC	(AAT)_5_	CDS	215–221	56	transcription factor TCP4-like [*Erigeron canadensis*]; XP_043628991.1
PX219834	EST-HiUP-11	F: GCGGATTGATGTCCATGCAC R: GTCGACCATGATGATCGCCT	(ATC)_7_	CDS	155–158	56	transcription factor MYB46 [*Helianthus annuus*]; XP_022026403.1
PX219835	EST-HiUP-12	F: TCGAAGAAGCTGCGAGGAAT R: TCTCCATGTTTCCCCATTCCA	(GAT)_14_	CDS	181–211	62, 64 **	protein BIG GRAIN 1-like B [*Lactuca sativa*]; XP_023761934.1
PX219836	EST-HiUP-13	F: ACCTCCTTTGCCACTTGGAG R: GGAGGCAACATGGTACCCAA	(CCT)_5_	CDS	131–137	60	protein EARLY FLOWERING 5 [*Erigeron canadensis*]; XP_043614864.1
PX219837	EST-HiUP-14	F: AAGTATCCCTCAACAGCGCG R: ACCGCAATAGCCTTTCCCTC	(TGA)_7_	CDS	147–156	62	AAA-ATPase ASD, mitochondrial-like [*Erigeron canadensis*]; XP_043628871.1
PX219838	EST-HiUP-15	F: AAGCGATGTCTACTGCGTGG R: TTCGGTACAAGCAGCTCCAA	(ATC)_6_	CDS	231–237	56	pollen receptor-like kinase 3 [*Cynara cardunculus* var. *scolymus*]; XP_024978884.1
PX219839	EST-HiUP-16	F: TGGAGCCAATTCAAGATCGGA R: TGCTCGAGTTTCTCCCATGT	(ATG)_5_	CDS	187–190	56	F-box/LRR-repeat MAX2 homolog A [*Erigeron canadensis*]; XP_043632012.1
PX219840	EST-HiUP-17	F: CCTACTCTGCAGATGAGGCC R: CCATATCACTGCAGCGCCTA	(AGA)_6_	CDS	133–136	56	metal tolerance protein 1 [*Helianthus annuus*]; XP_022025519.1
PX219841	EST-HiUP-18	F: GGTCGTTTCGTTCAAAGGCC R: GCGTACCATGACTTTGGCCT	(TCC)_5_	CDS	214–220	56, 58 **	glycerol-3-phosphate 2-O-acyltransferase 6 [*Cynara cardunculus* var. *scolymus*]; XP_024981596.1
PX219842	EST-HiUP-19	F: GAAGACGTGATTGAGCCCCA R: AACTCCCGATTTCGACACCC	(GGT)_6_	CDS	190–208	60	mechanosensitive ion channel protein 8-like isoform X3 [*Cynara cardunculus* var. *scolymus*]; XP_024961283.1
PX219843	EST-HiUP-20	F: AAACGGAGCAGCCAGATGAA R: CATCAGTTCCATCCCCAGCA	(TGA)_6_	CDS	208–211	56	glucosidase 2 subunit beta isoform X3 [*Helianthus annuus*]; XP_022009304.1
PX219844	EST-HiUP-21	F: CGTGTGGCACAAGAGTTAGC R: ATTCCCTTCGCCAATCCTGG	(TGA)_7_	CDS	236–245	56, 60 **	transcription elongation factor SPT6-like [*Erigeron canadensis*]; XP_043635655.1
PX219845	EST-HiUP-22	F: AACTTGTGACGGGGAGAAGG R: CTCATGCTCGGCCGTAGATT	(ATG)_5_	CDS	225–228	56	receptor protein-tyrosine kinase CEPR2 [*Cynara cardunculus* var. *scolymus*]; XP_024994229.1
PX219846	EST-HiUP-23	F: ATTGTCAGAGCACCAGCCTC R: GGTGGAGGAGGAGCTACAGA	(CCG)_5_	CDS	116–126	56	protein CHUP1, chloroplastic [*Cynara cardunculus* var. *scolymus*]; XP_024988773.1

* Primers elongated for M13 (-21) 18 bp sequence (5′-TGTAAAACGACGGCCAGT-3′) at their 5′ ends. ** Adjusted optimized annealing temperature in case of multiple alleles (sample specific).

**Table 6:** Characteristics of twelve EST-SSR markers for 270 *H. italicum* samples from Corsica and the Istrian peninsula (N_a_—no. of different alleles per locus; N_e_—no. of effective alleles per locus; H_o_—observed heterozygosity; H_e_—expected heterozygosity; PIC—polymorphic information content; I—Shannon’s information index; HWE—probability deviation from Hardy—Weinberg equilibrium; F_(null)_—estimated frequency of null alleles).

Locus	N_a_	N_e_	H_o_	H_e_	PIC	I	HWE	F_(null)_
EST-HiUP-01	8	3.66	0.619	0.727	0.690	1.529	**	0.0759
EST-HiUP-02	5	2.11	0.944	0.525	0.415	0.820	***	−0.2947
EST-HiUP-03	10	5.57	0.759	0.820	0.796	1.843	NS	0.0391
EST-HiUP-06	6	1.35	0.256	0.261	0.250	0.588	NS	0.0068
EST-HiUP-07	7	1.85	0.430	0.460	0.442	1.022	NS	0.0231
EST-HiUP-10	5	1.46	0.296	0.317	0.296	0.633	NS	0.0363
EST-HiUP-12	10	4.76	0.767	0.790	0.761	1.770	NS	0.0149
EST-HiUP-13	5	2.60	0.970	0.615	0.546	1.092	***	−0.2591
EST-HiUP-14	6	2.38	1.000	0.580	0.494	1.043	***	−0.2906
EST-HiUP-18	7	2.18	0.548	0.542	0.515	1.159	NS	−0.0081
EST-HiUP-19	8	4.90	0.789	0.796	0.766	1.744	NS	0.0044
EST-HiUP-21	6	1.72	0.478	0.419	0.393	0.868	*	−0.0828
Mean	6.92	2.88	0.655	0.571	0.530	1.176		

^NS^ Not significant. * Statistically significant difference *p* < 0.05. ** Statistically significant difference *p* < 0.01. *** Statistically significant difference *p* < 0.001.

**Table 7:** Genetic variability analysis of the *H. italicum* populations based on 12 EST-SSR markers (N–no. of different alleles per population; N_a_—average no. of different alleles per population; N_e_—no. of effective alleles per population; H_o_—observed heterozygosity; H_e_—expected heterozygosity; I—Shannon’s information index; F—inbreeding coefficient; No. of private alleles—number of alleles unique to a single population).

Population	N	N_a_	N_e_	H_o_	H_e_	I	F	No. of Private Alleles *
Capo Pertusato	45	3.75	2.29	0.601	0.490	0.901	−0.217	0
Plage de l’Ovu Santu	51	4.25	2.22	0.604	0.493	0.930	−0.227	3
Conca	55	4.58	2.73	0.700	0.562	1.072	−0.277	1
Punta di a Vacca Morta	46	3.83	2.63	0.708	0.548	1.005	−0.279	0
Col de Bavella	50	4.17	2.71	0.694	0.555	1.041	−0.243	0
Col de Saint-Eustache	56	4.67	2.82	0.742	0.598	1.129	−0.260	1
Ajaccio	45	3.75	2.54	0.656	0.518	0.947	−0.223	0
Corte	50	4.17	2.35	0.592	0.477	0.909	−0.206	0
Sagone	52	4.33	2.61	0.663	0.550	1.043	−0.225	0
Piana	52	4.33	2.63	0.692	0.549	1.047	−0.270	2
Pianottoli	44	3.67	2.19	0.650	0.490	0.878	−0.297	0
Tonnara	49	4.08	2.42	0.567	0.501	0.948	−0.104	2
Cape Kamenjak	48	4.00	2.50	0.641	0.519	0.976	−0.243	1

* Private alleles found in 13 populations of *H. italicum*, not including alleles from *H. litoreum* and *H. arenarium*.

**Table 8:** Analysis of molecular variance (AMOVA) across 13 populations of *H. italicum* using 12 EST-SSRs (df—degrees of freedom; SS—sum of squares; MS—mean square deviations; Est. Var.—estimated variance component; %—percentage of total variance).

Source	df	SS	MS	Est. Var.	%
Among populations	12	285.650	23.804	0.904	15%
Within populations	257	1294.131	5.036	5.036	85%
Total	269	1579.781		5.940	100%

**Table 9:** Population genetic parameters of the 4 *H. italicum* genetic clusters based on STRUCTURE assignment with K = 4 (N_pop_—no. of populations included; H_o_—observed heterozygosity; H_e_—expected heterozygosity; N_pr_—no. of private alleles; N_ar_—allelic richness; N_par_—private allelic richness).

Genetic Cluster (K)	N_pop_	H_o_	H_e_	N_pr_	N_ar_	N_par_
1	1	0.583	0.492	1	3.010	0.330
2	3	0.684	0.555	4	3.360	0.330
3	5	0.669	0.576	3	3.560	0.390
4	4	0.637	0.552	7	3.470	0.420

**Table 10:** Sampling locations of *H. italicum* and sample sources for two other studied *Helichrysum* species (*H. arenarium* and *H. litoreum*).

Sampling Location	Species	Number of Samples
France, Corsica, Capo Pertusato	*H. italicum*	19
France, Corsica, Col de Bavella	*H. italicum*	21
France, Corsica, Col de Saint-Eustache	*H. italicum*	20
France, Corsica, Conca	*H. italicum*	20
France, Corsica, Plage de l’Ovu Santu	*H. italicum*	20
France, Corsica, Punta di a Vacca Morta	*H. italicum*	20
France, Corsica, Corte	*H. italicum*	20
France, Corsica, Ajaccio	*H. italicum*	23
France, Corsica, Sagone	*H. italicum*	21
France, Corsica, Piana	*H. italicum*	20
France, Corsica, Pianottoli	*H. italicum*	20
France, Corsica, Tonnara	*H. italicum*	20
Croatia, Istria, Cape Kamenjak	*H. italicum*	26
Slovenia, commercially available *H. arenarium* tea (Flora Ltd., Rogatec, Slovenia)	*H. arenarium*	6 bulk samples
Slovenia, plants from purchased seeds, grown in an ex situ collection of UP FAMNIT	*H. litoreum*	6

## Data Availability

Raw sequences were submitted to the NCBI Short Read Archive (SRA) with the accession number PRJNA1308477. Sequences of loci with EST-SSRs and primers were submitted to NCBI Nucleotide database with accession numbers from PX219824 to PX219846. The original contributions presented in the study are included in the article/Supplementary Material. Further inquiries can be directed to the corresponding author.

## Supplementary Materials

The following supporting information can be downloaded at https://www.mdpi.com/article/10.3390/plants14243794/s1: Table S1: Comparison of the 88 amplified alleles (in bp) in transferability testing of 12 newly developed EST-SSRs to *H. italicum*, *H. litoreum*, and *H. arenarium* (green–allele present; white–allele not present); Table S2: *H. italicum* populations with private alleles per loci and its frequency; and Table S3: Matrix of pairwise population F_st_ analysis (F_st_ values below diagonal).
