# Supplementary material for: First EST-SSRs of Helichrysum italicum (Roth) G. Don (Asteraceae) Revealed Insights into the Genetic Diversity and Population Structure in Corsica
Source: Plants (Basel). 2025 Dec 12;14(24):3794. doi: 10.3390/plants14243794 (PMC12737098; doi:10.3390/plants14243794)
Supplement: Supplementary file 1 [file plants-14-03794-s001.zip › plants-3976450-supplementary.pdf]

Supplementary Materials

Table S1. Comparison of the 88 amplified alleles (in bp) in transferability testing of 12 newly developed EST-SSRs to *H. italicum*, *H. litoreum* and *H. arenarium* (green–allele present; white–allele not present).

Locus	*Helichrysum italicum*	*Helichrysum litoreum*	*Helichrysum arenarium*	Allele length (bp)
EST-HiUP-01	green			195
	green			201
	green	green		204
	green	green		207
	green	green		210
	green		green	213
	green	green	green	216
	green	green	green	219
EST-HiUP-02	green	green	green	204
	green		green	207
	green			210
	green			213
	green			216
EST-HiUP-03		green	green	262
	green	green	green	264
	green		green	267
	green			270
	green		green	273
	green			276
	green			279
	green			282
	green			285
	green			289
	green			295
EST-HiUP-06	green			165
	green	green		168
	green	green	green	171
	green	green		174
	green	green		177
	green			188
EST-HiUP-07	green			244
	green	green	green	246
	green		green	248
	green		green	250
	green			252
	green			254
	green			256
EST-HiUP-10	green			208
	green			215
	green	green	green	218
	green	green		221
	green		green	224
			green	227
			green	230
			green	232

EST-HiUP-12				181
				184
				187
				190
				193
				196
				199
				202
				205
				208
				211
EST-HiUP-13				128
				131
				134
				137
				140
EST-HiUP-14				147
				150
				153
				156
				159
				162
EST-HiUP-18				208
				211
				214
				217
				220
				223
				226
EST-HiUP-19				193
				196
				199
				202
				205
				208
				211
				214
EST-HiUP-21				233
				236
				239
				242
				245
				248

Table S2. *H. italicum* populations with private alleles per loci and its frequency.

Population	Locus	Allele length	Frequency
Plage de l'Ovu Santu	EST-HiUP-03	295	0.025
Plage de l'Ovu Santu	EST-HiUP-10	208	0.050
Plage de l'Ovu Santu	EST-HiUP-21	233	0.025
Piana	EST-HiUP-03	285	0.025
Piana	EST-HiUP-06	188	0.025
Tonnara	EST-HiUP-13	128	0.050
Tonnara	EST-HiUP-14	162	0.125
Conca	EST-HiUP-01	195	0.025
Col de Saint-Eustache	EST-HiUP-07	244	0.025
Cape Kamenjak	EST-HiUP-12	211	0.019

Table S3. Matrix of the pairwise population F_{st} analysis (F_{st} values below diagonal).

	Capo Pertusato	Plage de l'Ovu Santu	Conca	Punta di a Vacca Morta	Col de Bavella	Col de Saint-Eustache	Ajaccio	Corte	Sagone	Piana	Pianottoli	Tonnara	Cape Kamenjak	
**Capo Pertusato**	0													**Capo Pertusato**
**Plage de l'Ovu Santu**	0.045	0												**Plage de l'Ovu Santu**
**Conca**	0.057	0.023	0											**Conca**
**Punta di a Vacca Morta**	0.050	0.018	0.014	0										**Punta di a Vacca Morta**
**Col de Bavella**	0.056	0.021	0.017	0.021	0									**Col de Bavella**
**Col de Saint-Eustache**	0.061	0.030	0.013	0.021	0.018	0								**Col de Saint-Eustache**
**Ajaccio**	0.062	0.061	0.048	0.057	0.044	0.055	0							**Ajaccio**
**Corte**	0.056	0.027	0.040	0.039	0.032	0.047	0.051	0						**Corte**
**Sagone**	0.056	0.038	0.039	0.039	0.031	0.037	0.04	0.019	0					**Sagone**
**Piana**	0.042	0.024	0.029	0.027	0.026	0.027	0.047	0.022	0.015	0				**Piana**
**Pianottoli**	0.049	0.055	0.052	0.054	0.056	0.048	0.066	0.067	0.061	0.043	0			**Pianottoli**
**Tonnara**	0.039	0.048	0.046	0.047	0.048	0.047	0.062	0.056	0.055	0.042	0.032	0		**Tonnara**
**Cape Kamenjak**	0.049	0.039	0.048	0.043	0.038	0.042	0.052	0.028	0.028	0.031	0.067	0.046	0	**Cape Kamenjak**
	**Capo Pertusato**	**Plage de l'Ovu Santu**	**Conca**	**Punta di a Vacca Morta**	**Col de Bavella**	**Col de Saint-Eustache**	**Ajaccio**	**Corte**	**Sagone**	**Piana**	**Pianottoli**	**Tonnara**	**Cape Kamenjak**	
